# Study of continuous flow ultrasonication to improve total phenolic content and antioxidant activity in sorghum flour and its comparison with batch ultrasonication

**DOI:** 10.1016/j.ultsonch.2020.105402

**Published:** 2020-12-03

**Authors:** Umesh C. Lohani, K. Muthukumarappan

**Affiliations:** Ag & Biosystems Engineering Deptt, South Dakota State University, Brooking, SD, USA

**Keywords:** Sorghum flour, Continuous flow ultrasonication, Batch ultrasonication, Total phenolic content, Antioxidant activity, Specific energy

## Abstract

Ultrasonic technology was applied to release the phenolics bound with starch and protein matrix in order to enhance total phenolic content (TPC) and antioxidant activity (AA) of the sorghum flour. Both the continuous flow and batch ultrasonication were implied with independent variables such as flour to water ratio (FWR), ultrasonication intensity (UI), and ultrasonication time (UT) with an additional variable as flow rate (FR) in continuous flow ultrasonication. All the process variables showed a significant effect on the corresponding ultrasonication process. The optimal conditions for the continuous flow ultrasonication were a FWR of 10% w/v, an UI of 20 W/cm^2^, an UT of 130 s, and 15 ml/s FR which produced a maximum values of 70.9 mg GAE/100 g dry matter (d.m.) for TPC and 143.9 µmol TE/100 g d.m. for AA. Regarding the batch ultrasonication, the maximum values were 65.6 mg GAE/100 g d.m. and 141.0 µmol TE/100 g d.m. for TPC and AA, respectively at optimum conditions of 10% w/v FWR, 30 W/cm^2^ UI, and 200 s UT. When comparing with the batch ultrasonication, the continuous flow process saved 35% time and 33% of energy consumption to obtain comparatively higher TPC and AA of the sorghum flour. Ultrasonication improved free phenolic acid content by releasing bound phenolics in the sorghum flour. Impact of various process parameters on specific energy was analyzed during both the processes, and influence of energy on TPC and AA of the sorghum flour was also observed for the batch and continuous flow ultrasonication.

## Highlights

• Ultrasonication was used to release the bound phenolics in sorghum flour.
• Continuous ultrasonication was more effective than batch process.
• Lower intensity and time required in continuous flow to obtain a specific energy.
• Less energy and time required to release more phenolics during continuous flow.

## Graphical abstract

**Figure:**
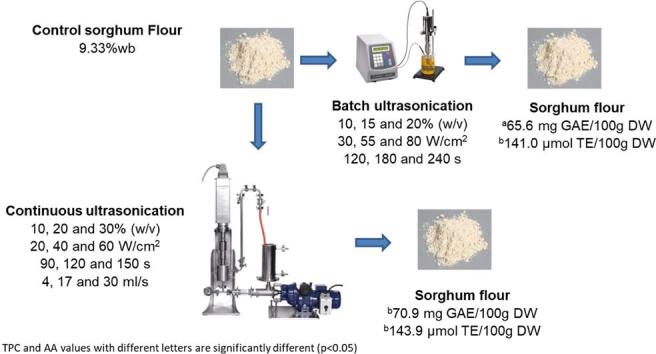

## 1 Introduction

In recent years, the research areas to develop the ready to eat food or snacks with higher antioxidant activity have been increased [1], [2], [3], [4]. Antioxidant rich foods have potential role in prevention or delaying atherosclerosis, heart disease, cerebrovascular, stroke, diabetes mellitus rheumatoid arthritis, osteoporosis, ulcers, sunburn, cataracts, and aging [5], [6]. Sorghum (*Sorghum bicolor* L.) is one of the crops that contains more diverse and abundant phenolic compounds mainly in the forms of phenolic acids and flavonoids compared to other major cereal crops [7], [8]. These compounds have potentiality to impact positively on human health because of their antioxidant and antiradical properties [9]. Sorghum utilization can be improved by incorporating it into mainstream human diet in different innovative ways such as extrusion and baking. Most of the phenolic compounds in plants are present in bound forms with carbohydrates, lignin, pectin and proteins [10], [11]. This bound nature of phenolics as glycosides reduces their ability to function as good antioxidants. Therefore, by liberating these bound phenolics using some pretreatments, antioxidants rich sorghum flour can be introduced to human diet.

In past few years, ultrasound assisted extraction of phenolic compounds from pomegranate peel [12], mustard [13], carrot pomace [14], grape pomace [15], beans [16], buckwheat [17], black soybean [18], and orange pomace [19] has been extensively investigated but use of ultrasonication to enhance the phenolics itself in food is limited [20], [21], [22] and is few in case of sorghum [23], [24]. The mechanism for ultrasonic is the cavitation of bubbles upon the propagation of the acoustic waves. Collapse of bubbles may induce physical, chemical, and mechanical effects, which results in the disruption of biological cell walls to facilitate the release of extractable compounds and thus increases the total phenolics and antioxidant activity. Ultrasonication separates starch from protein matrix and breaks down these molecules [25] resulting in releasing of bound phenolics with protein and other components.

Comparison of both the ultrasonication processes is required to ensure the analogy in treatment when switch from batch to continuous flow mode. Furthermore, comparison is also required to know the main factor which mostly influences the response parameters in the corresponding ultrasonication mode. Even though batch ultrasonication has been proven to be effective in extracting the phenolics in food materials, it undergoes the extraction difficulty for highly concentrated samples [21], [24]. From the earlier research, it could be hypothesized that higher sample concentration, less intensity and less time would be required in continuous flow ultrasonication to get the TPC and AA in the sorghum flour equivalent to that of batch ultrasonication [23], [24]. In addition to this, the influence of flow rate as an additional parameter on phenolic release in the continuous flow ultrasonication would be of interest. Therefore, based on these rationales, the objectives of the study were to understand the continuous flow ultrasonication behavior to enhance the TPC and AA in the sorghum flour and to compare it with the batch ultrasonication process.

## 2 Materials and methods

Sorghum flour (297 µm) provided by ADM Milling Co. (Overland Park, KS) was stored at −20 °C. For continuous flow process, ultrasonic device (UIP1000hd, Hielscher Inc., NJ, USA) with 20 kHz frequency, 1000 W power and a sonotrode of 22 mm tip diameter was used. The effective volume of flow cell after intruding the sonotrode was 165 ml. For the continuous flow ultrasonication, 100 ml distilled water was added to 10 g, 20 g and 30 g flour to keep the flour to water ratio (FWR) as 10%, 20%, and 30% (w/v). Sorghum slurry was then subjected to the ultrasonication at 20, 40, and 60 W/cm^2^ ultrasonication intensity (UI) for 90, 120, and 150 s. Total recirculation time was calculated for the whole sample to get it exposed to the probe for the corresponding ultrasonication time. Flow rate (FR) of slurry during ultrasonication was varied from 4 ml/s to 30 ml/s. Intensity was estimated by (P_s_-P_i_)/A, where P_s_ is the power delivered to the sample (W), P_i_ is the power delivered out of the sample at the same amplitude (W), and A is the surface area of the probe (cm^2^) [26], [27].

Ultrasonic processor (VC 505, Sonics and Materials Inc., CT, USA) with 20 kHz frequency, 500 W power and a sonotrode of 13 mm tip diameter was used for the batch ultrasonication. Sorghum flour was batch ultrasonicated for 10%, 15%, and 20% (w/v) FWR at 30, 55, and 80 W/cm^2^ intensity for 120, 180, and 240 s ultrasonication time (UT). The experimental range of all the process parameters for both the ultrasonication processes were decided based on the preliminary trials.

Ultrasonicated samples were oven dried at 40 °C till their constant weight and stored at −20 °C for TPC and AA analysis. Sample volume of 100 ml and 2000 ml were taken for the batch and continuous flow ultrasonication, respectively. Untreated sorghum flour was taken as a control for comparison. Specific energy (kJ/kg), the energy input per unit solid mass, transferred from ultrasound equipment to the sample was calculated using following equation [28], [29], [30], [31].

$$\mathrm{Specific}\;energy\;\left(kJ/kg\right)=\frac{\mathrm{Ultrasonication}\;power\;(kW)\times ultrasonication\;time(s)}{\mathrm{Sample}\;mass(kg)}$$

### 2.1 Preparation of samples for analysis of TPC and AA

For determining TPC and AA, 1 g of control or ultrasonicated sorghum flour was mixed with 10 ml of methanol followed by shaking at low speed for 1 h and then centrifuged at 3000×g for 20 min. The supernatant was decanted and the residue was re-extracted as described above. The two supernatants were combined and stored at −20 °C until analysis for TPC and AA.

Free phenolic acid extraction was performed by adding 10 ml of 80% (v/v) aqueous methanol into 2 g of control or ultrasonicated sorghum flour. Mixture was shaken in a shaking water bath for 1 h at 25 °C. After centrifugation at 3000×g for 20 min, the supernatant was decanted and the extraction was repeated as described above. The two supernatants were combined, evaporated to near dryness and reconstituted with methanol to a final volume of 10 ml [32].

#### 2.1.1 Total phenolic content (TPC)

TPC of the sample was determined using Folin–Ciocalteu method [33]. Data were expressed in mg Gallic acid equivalent (GAE) per 100 g dry matter (d.m.). This analysis was done in six replications.

#### 2.1.2 Antioxidant activity (AA)

Extinction of DPPH is a free radical scavenging activity which was measured using spectrophotometric method described by Brand-Williams, Cuvelier and Berset [34]. Results were expressed as µmol trolox equivalent (TE) per 100 g d.m. Samples were analyzed in six replications.

#### 2.1.3 Free phenolic acid characterization

Analysis of sample extracts was carried out using Thermo Scientific, Dionex Ultimate 3000 UHPLC system (Bannockburn, IL, United States) equipped with diode-array detector (DAD) and C_18_ column (150 mm × 4.6 mm) packed with 5 μm particles. The samples were injected with a mobile-phase flow rate of 800 μL/min. Gradient elution was carried out with a solvent system of water/acetic acid (99.8:0.2 v/v) as mobile phase A and acetonitrile/acetic acid (99.8:0.2 v/v) as mobile phase B. The total run time was 12 min, and the gradient elution was as follows: 0.0–3.0 min, B 10–25%; 3.0–4.5 min, B 25–45%; 4.5–6.5 min, B 45–65%; 6.5–8.0 min, B 65–85%; 8.0–9.0 min, B 85–100%.; 9.0–12.0 min, B 100–10%. All the solvents were filtered through 0.22 μm PTFE filters prior to inject. The column was maintained at 30 °C while the autosampler was thermostated at 4 °C. The system was controlled by Thermo Scientific Dionex Chromeleon 7 software. Benzoic acid and cinnamic acid derivatives were detected at 280 nm and 320 nm, respectively. The concentrations of phenolic acids were calculated from peak areas in comparison to calibration curves of the respective standards and were expressed as μg/g d.m.

### 2.2 Total starch, protein, crude fiber and moisture content

Protein, crude fiber and moisture content of control, continuous flow and batch ultrasonicated sorghum flour were analyzed using AOAC [35]. Total starch was determined by AOAC approved method 996.11 [36].

### 2.3 Experimental design

A Box-Behnken design was applied on both the methods to determine the effects and the optimum levels of the process parameters [17], [37], [38], [39]. The effects were studied at three experimental levels –1, 0, and +1. A total of 30 and 17 experiments were required for continuous flow and batch ultrasonication, respectively. The experimental data were analyzed by the response surface regression procedure and the parameters obtained from the response surface methodology (RSM) analysis were substituted into the following second-order polynomial model equation.

$$Y_{i}=\beta_{0}+\sum_{i=1}^{k}\beta_{i}Xi+\sum_{i=1}^{k}\beta_{\mathrm{ii}}X_{\mathrm{ii}}^{2}+\sum_{i=1}^{k}\sum_{j=i+1}^{k}\beta_{\mathrm{ij}}X_{i}X_{j}$$

where Y_i_ is the predicted response; β_0_ is the interception coefficient; β_i_, β_ii_, and β_ij_ are coefficients of the linear, quadratic, and interaction terms; X_i_ and X_j_ are the variables; and k is the number of independent parameters (k = 4 and 3 for continuous flow and batch ultrasonication, respectively).

### 2.4 Statistical analysis

Design expert 9 statistical software package (Stat-Ease Inc., USA) was used to analyze the experimental data. Multiple regression analysis and analysis of variance (ANOVA) were used to evaluate the experimental data. The adequacy and quality of the models were examined by evaluating the lack of fit (LOF), the coefficient of determination R^2^, adjusted R^2^, predicted R^2^, coefficient of variance and the Fisher test value (F-value) obtained from the ANOVA. Derringer’s desired function methodology was used to generate optimal conditions for continuous flow (FWR, UI, UT and FR) and batch ultrasonication (FWR, UI and UT) on the TPC and AA of the sorghum flour.

## 3 Results and discussion

### 3.1 Model fitting

The response surface methodology approach was conducted to determine the effect of continuous flow and batch ultrasonication on TPC and AA of the sorghum flour. For continuous flow ultrasonication, the observed values of TPC and AA were found in range of 52.8–69.9 mg GAE/100 g d.m. and 91.4–143.3 µmol TE/100 g d.m., respectively. The observed values ranged from 36.1 to 66.0 mg GAE/100 g d.m. for TPC and 89.8 to 140.4 µmol TE/100 g d.m. for AA in case of batch ultrasonication. Among the experimental process conditions, the highest TPC and AA were obtained at 10% (w/v) FWR, 20 W/cm^2^ UI, 90 s UT, and 17 ml/s FR for continuous flow ultrasonication, whereas, for batch ultrasonication, 10% (w/v) FWR, 30 W/cm^2^ UI, and 240 s UT were obtained for maximum TPC and AA of the sorghum flour.

The regression coefficients of mathematical model analyzed by RSM describing the TPC and AA of the sorghum flour as a function of selected process parameters for continuous flow and batch ultrasonication are depicted in Table 1. The analysis of variance is also summarized to show the significance of the regression coefficients, the goodness of fit, and the adequacy and quality of the models.

**Table 1:** Regression coefficients and statistical parameters describing the effect of the independent variables on TPC and AA of sorghum flour for continuous and batch ultrasonication.

Model term	Regression coefficient estimated				
	Continuous flow ultrasonication			Batch ultrasonication	
	TPC	AA		TPC	AA
β_0_	10.4***	112.4***	β_0_	85.1***	65.9***
β_1_	0.61***	−1.66***	β_1_	−2.28***	0.79***
β_2_	−0.08***	−1.13***	β_2_	−0.89***	−0.07***
β_3_	0.85***	0.87***	β_3_	0.21***	0.83***
β_4_	0.41***	0.48***	β_11_	0.05*	−0.09**
β_11_	−0.02***	0.01*	β_22_	0.005***	−0.005**
β_22_	−0.005***	0.005***	β_33_	−0.0004*	−0.002***
β_33_	−0.003***	−0.003***	β_12_	−0.003	−0.002
β_44_	−0.02***	−0.02***	β_13_	−0.0001	−0.0003
β_12_	0.0001	−0.001	β_23_	−0.00006	0.00004
β_13_	−0.002	0.0001			
β_14_	−0.001	−0.003			
β_23_	0.001	−0.0001			
β_24_	0.007***	0.002			
β_34_	0.0005	0.001			

Adequacy of mathematical model					
p (Lack of fit)	0.68	0.98		0.79	0.99
R^2^	0.98	0.99		0.99	0.99
R^2^_adj_	0.96	0.98		0.97	0.98
R^2^_perd_	0.92	0.97		0.95	0.99
CV (%)	1.51	1.58		2.87	1.56
p (F value)	0.000	0.000		0.000	0.000

Significant at *p < 0.1, **p < 0.05, ***p < 0.0.01, CV: Coefficient of Variance, adj: adjusted, pred: predicted

The values of the regression coefficients presented in Table 1 were used in the final predictive model equations after discarding the non-significant parameters. Thus, these equations were assumed to best describe the relationships between the experimental variables and the response factors.

### 3.2 Interpretation of response surface model and contour plots

Three-dimensional response surface plots and two-dimensional contour plots were obtained based on the model equations mentioned above to explicate the correlation between independent and dependent variables. Both type of plots presented the effects of two independent variables on the response factor, keeping others at level-coded zero.

#### 3.2.1 Effect of ultrasonication variables on TPC of sorghum flour

**Fig. 1:**
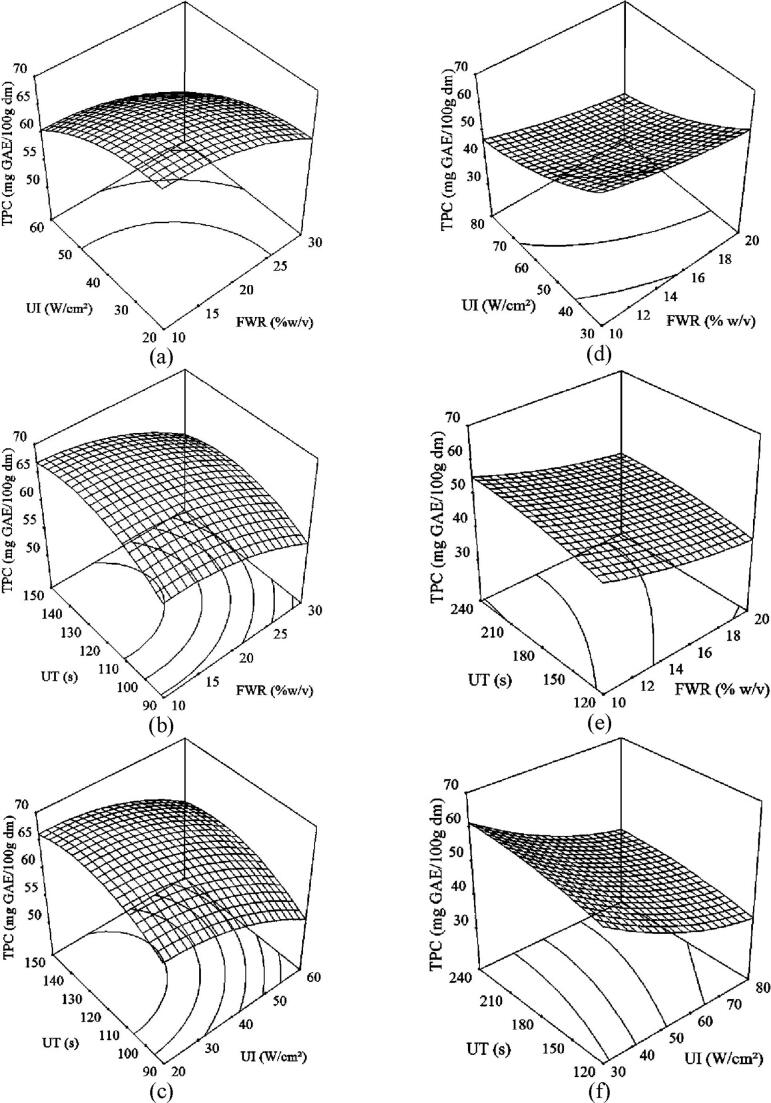
Response surface plots of total phenolic content of sorghum flour as affected by flour to water ratio, ultrasonication intensity, and ultrasonication time for (a, b, c) continuous flow and (d, e, f) batch ultrasonication at 0 level of corresponding third variable.

The effect of FWR, UI, and UT on TPC of the sorghum flour for the continuous flow and batch ultrasonication is shown in Fig. 1. With regard to the combined effect by FWR and UI for the continuous flow ultrasonication (Fig. 1a), maximum TPC was obtained in the sorghum flour up to 25% (w/v) FWR at low UI (20 W/cm^2^), whereas TPC started decreasing after 15% (w/v) FWR during the batch ultrasonication at low UI (30 W/cm^2^) (Fig. 1d). This result might be attributed to the early stage agitation instead of cavitation with the effect of UI in the batch process when bubble cloud density became too large resulted in rise to shielding effects, coalescence and general bubble–bubble interactions that decreased the overall cavitation efficiency of the process [40], [41]. TPC decreased gradually by 12% with increase in FWR from 10% to 30% (w/v) at higher UI (60 W/cm^2^) for the continuous flow process (Fig. 1a). Similar result was obtained for the batch process at higher UI (80 W/cm^2^) with increment in FWR from 10% to 20% (w/v) (Fig. 1d). Although, the maximum TPC was obtained at the low ultrasonication intensity with lower concentration of sample for both the processes, continuous flow ultrasonication released 8% more TPC in the sorghum flour at 33% less UI and for 67% more FWR. These results were in contrast with the findings of Carrera, Ruiz-Rodríguez, Palma and Barroso [42], González-Centeno, Knoerzer, Sabarez, Simal, Rosselló and Femenia [43] and Pan, Qu, Ma, Atungulu and McHugh [12] who observed an increase in TPC of grape, grape pomace and pomegranate peel, respectively with an increase in ultrasound power. Carrera, Ruiz-Rodríguez, Palma and Barroso [42] and Tabaraki and Nateghi [44] also reported a reduction or no significant change in TPC of grape and rice bran, respectively with an increase in the sample concentration in solvent. With an increase in FWR, the viscosity of solution increased and because of that the ultrasound energy might not be transmitted uniformly to the whole solution at a given ultrasonication intensity [21]. The lower the flour to water ratio, the greater the driving force within the solid resulted in increase of diffusion rate [45], [46]. The main effect of the FWR was to modify the solubility and equilibrium constants and thus increased the TPC to a maximum at the lowest FWR.

The trend observed for TPC of the sorghum flour upon simultaneous variation of FWR and UT is exhibited in Fig. 1b, e for the continuous flow and batch ultrasonication, respectively. At low FWR (10 w/v), the continuous flow process produced sorghum flour with maximum TPC in 110 s, whereas maximum TPC was obtain in 210 s during the batch ultrasonication. Stagnant sample in the batch process might cause the accumulation of acoustic energy near the probe which transmitted slowly in the whole sample resulted in more input of energy [47]. On the other hand, continuous flow of sample might allow the transmittance of acoustic energy efficiently and uniformly in the whole sample. With increase in FWR from 10 to 30% (w/v), TPC of the sorghum flour during continuous flow process depleted by 9% regardless the UT (Fig. 1b), whereas for the batch process, TPC decreased by 19% with increase in FWR from 10 to 20% (w/v) regardless the UT (Fig. 1e). Even though, low FWR and higher duration indicated the maximum TPC in the sorghum flour for both the processes, continuous flow ultrasonication provided 20% more TPC at 91% less time with compared to the batch ultrasonication. These findings are in agreement with the Carrera, Ruiz-Rodríguez, Palma and Barroso [42] and Jabbar, Abid, Wu, Hashim, Saeeduddin, Hu, Lei and Zeng [14] who also reported insignificant change in TPC of grapes and carrot pomace, respectively at longer extraction time.

Finally, the plot of TPC as affected by UI and UT for the continuous flow and batch ultrasonication are shown in Fig. 1c, f, respectively. For the continuous flow process, TPC increased by 3% when UT increased from 90 s to 100 s followed by insignificant (p > 0.05) change in TPC at low UI (20 W/cm^2^) (Fig. 1c). However, for the batch process, UT didn’t have any significant (p > 0.05) effect on TPC at low UI (30 W/cm^2^). Having said that, TPC gradually increased by 6% with an increase in UT from 120 s to 240 s at higher UI (80 W/cm^2^) (Fig. 1f). Low UI in the batch ultrasonication might not provide enough acoustic energy in the standstill sample to get transmitted into the whole sample [48]. Both the processes exhibited a declined trend in TPC with an increase in UI regardless the UT though the effect of UI on TPC was observed more severe for the batch ultrasonication (Fig. 1c and f). Though both the processes exhibited the higher TPC in the sorghum flour at low UI, the continuous flow ultrasonication released 8% more TPC at 33% less UI consuming 43% less time with compared to the batch process.

**Fig. 2:**
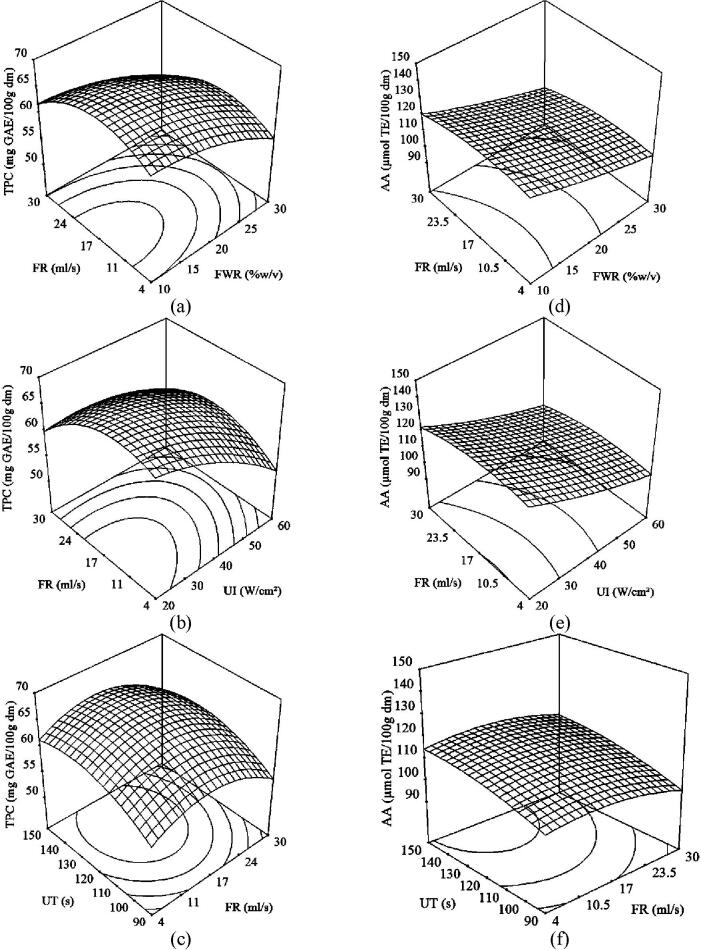
Response surface plots of total phenolic content (a, b, c) and antioxidant activity (d, e, f) of sorghum flour as affected by combination of flour to water ratio, ultrasonication intensity and ultrasonication time with FR for continuous flow ultrasonication at 0 level of corresponding third variable.

The response surface of the effect of FR with FWR, UI and UT for the continuous flow ultrasonication is shown in Fig. 2. Maximum TPC was obtained at low FWR (10% w/v) (Fig. 2a) and low UI (20 W/cm^2^) (Fig. 2b) with moderate values of FR ranged from 15 to 20 ml/s. Fig. 2c exhibits the combined effect of FR and UT on TPC. As observed, a gradual increase of UT up to 130 s resulted in increased TPC by 10% at a FR level of 15–17 ml/s, followed by an insignificant change. Lower flow rate might cause the overheating of the sample due to an elongation in sample probe contact time for each cycle [49]. On the other hand, the sample-probe contact time during higher flow rate was too short for each cycle to transmit the acoustic energy to the sample resulted in less cavitation.

#### 3.2.2 Effect of ultrasonication variables on AA of sorghum flour

**Fig. 3:**
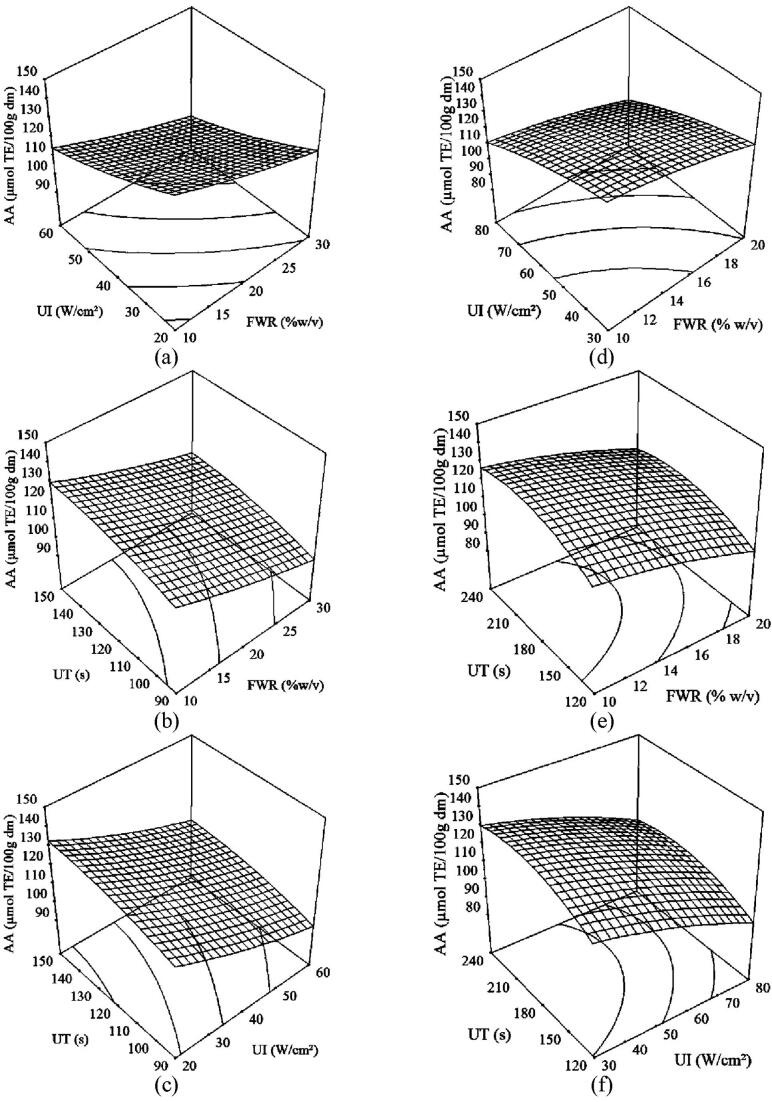
Response surface plots of antioxidant activity of sorghum flour as affected by flour to water ratio, ultrasonication intensity, and ultrasonication time for (a, b, c) continuous flow and (d, e, f) batch ultrasonication at 0 level of corresponding third variable.

As explicated in response surface plots for AA (Fig. 3), the ultrasonication variables for the continuous flow and batch process, i.e. FWR, UI and UT affected the response factors in a way similar to that observed for the TPC. These results supported the claims that the AA of the plant extracts is associated substantially with their TPC.

AA decreased significantly (p < 0.05) with an increase in FWR and UI for both continuous flow (Fig. 3a) and batch (Fig. 3d) ultrasonication. Both the response surface plots also show that this effect of one variable was regardless of other. Even though maximum AA was obtained at low FWR and UI for both the ultrasonication processes, continuous flow exhibited comparatively 7% more AA in the sorghum flour at 33% less UI. Similar results were found by Gribova et al. [50] for AA of bearberry leaves. Tabaraki and Nateghi [44] also reported no significant change in scavenging activity of DPPH of rice bran at higher liquid to solid ratio. Free radicals might be produced with an increase in UI, which inclined to reduce the antioxidant activity in the sorghum sample [51].

For the continuous flow ultrasonication, AA of the sorghum flour gradually increased by 10% with an increase in UT from 90 s to 150 s at higher FWR (30% w/v). This relation was less effective at low FWR (10% w/v) though there was no significant (p > 0.05) increase in AA at higher level of UT (150 s) (Fig. 3b). Similar results were observed for the batch ultrasonication where maximum AA was found at low FWR when UT increased up to 130 s, followed by no change (Fig. 3e). These results are in accordance with the finding of Jabbar, Abid, Wu, Hashim, Saeeduddin, Hu, Lei and Zeng [14] and Pan, Qu, Ma, Atungulu and McHugh [12] who also reported no significant change in antioxidant capacity of carrot pomace and pomegranate peel, respectively at longer extraction time.

Fig. 3c, f shows the effect of UI and UT on AA of the sorghum flour for the continuous flow and batch ultrasonication, respectively. For both the processes, maximum AA was observed at low UI and higher UT, nevertheless, no significant change in AA was observed after 130 s of ultrasonication time during the continuous flow process (Fig. 3c). For the batch process, lower UI (30 W/cm^2^) favored increase in AA by 8% with increase in UT up to 180 s, followed by no change at all (Fig. 3f).

It is obvious from Fig. 3 that AA of the sorghum flour ranged from 140 to 143 µmol TE/100 g d.m. was observed at 33% and 28% less UI and UT, respectively during the continuous flow ultrasonication as compare to those of the batch process. This result was attributed to the amount of TPC presented in the sample.

Three dimensional plots for AA of the sorghum flour showing the effect of FR along with FWR, UI and UT in continuous flow ultrasonication are depicted in Fig. 2. AA was found maximum for the moderate FR values ranged from 15 to 20 ml/s at low FWR (10% w/v) and UI (20 W/cm^2^) when UT increased up to 130 s, followed by stability in AA data.

### 3.3 Optimization of the ultrasonication processes and validation

Optimal process conditions were investigated for the continuous flow and batch ultrasonication and to determine the maximum TPC and AA of the sorghum flour using Derringer’s desired function methodology. This algorithm varies on a scale of 0–1, where 0 represents a completely undesirable response, and 1 depicts the most desirable one. Specifically, the global desirability values of 0.98 and 0.97 were observed when optimum conditions were obtained for the continuous flow and batch ultrasonication by maximizing the response factors. Table 2 indicates the optimum conditions for both the ultrasonication process along with predicted and experimental TPC and AA values. The predicted results matched well with the experimental results which validated the RSM model, indicating Box-Behnken design could be effectively used to optimize the process parameters for both the ultrasonication processes on TPC and AA of the sorghum flour.

**Table 2:** Estimated optimum conditions, predicted and experimental values of responses for continuous and batch ultrasonication.

Ultrasonication	Optimum conditions				Response variables			
	FWR (%w/v)	UI (W/cm^2^)	UT (s)	FR (ml/s)	TPC, mg GAE/100 g d.m.		AA, µmol TE/100 g d.m.	
					Predicted	Actual	Predicted	Actual
Continuous flow	10	20	130	15	71.0	70.9 ± 1.79	144.7	143.9 ± 3.58
Batch	10	30	200	–	66.4	65.6 ± 1.45	141.7	141.0 ± 3.23

Response experimental results are reported as mean ± standard deviation (n = 6)

It is obvious from Table 2 that approximately 6% and 2% more TPC and AA, respectively were obtained using continuous flow ultrasonication as compare to the batch process. With compare to the control sorghum flour, continuous flow ultrasonicated sorghum flour had 11% and 7.9% more TPC and AA, respectively. Furthermore, corresponding to these results, continuous flow ultrasonication interestingly reduced 33% UI and 35% UT, providing less time and low energy consumption with compare to the batch ultrasonication. Pan, Qu, Ma, Atungulu and McHugh [12] reported that maximum phenolic content and antioxidant capacity from pomegranate peel were found at 59.2 W/cm^2^ ultrasound intensity during the continuous flow process.

Increase in phenolic content during ultrasonication was due to release of bound phenolics in the sorghum flour. Table 3 depicts that ultrasonicated sorghum flours had significantly (p < 0.05) more benzoic acid and cinnamic acid derived phenolic acids than that of the control sorghum flour. However, *p*-coumaric acid and salicylic acid were not significantly (p > 0.05) different in the control sorghum flour and batch ultrasonicated sorghum flour. It was also observed that total starch, crude protein and crude fiber of the control and ultrasonicated (at optimum conditions for the batch and continuous flow) sorghum flours varied from 72.4 to 73.0 g/100 g d.m., 11.1–11.9 g/100 g d.m. and 1.23–1.27 g/100 g d.m., respectively. It indicates that ultrasonication didn’t have any significant effect on starch, protein and fiber of the sample.

**Table 3:** Phenolic profile of continuous and batch ultrasonicated sorghum flours (μg/g DW).

Compounds	Control SF	Continuous flow ultrasonicated SF	Batch ultrasonicated SF
*Benzoic acids*			
Protocatechuic acid	6.18 ± 0.11^a^	7.11 ± 0.12^b^	6.74 ± 0.14^c^
*p*-Hydroxybenzoic acid	13.3 ± 0.22^a^	14.8 ± 0.29^b^	13.9 ± 0.21^c^

*Cinnamic acids*			
Caffeic acid	10.2 ± 0.19^a^	13.5 ± 0.17^b^	12.6 ± 0.23^c^
*p*-coumaric acid	4.87 ± 0.13^a^	5.53 ± 0.11^b^	4.94 ± 0.11^a^
Ferulic acid	13.4 ± 0.28^a^	16.7 ± 0.21^b^	14.9 ± 0.19^c^
Salicylic acid	22.8 ± 0.20^a^	24.5 ± 0.18^b^	22.5 ± 0.15^a^

Means in the same row with different letters are significantly different (p < 0.05), SF: sorghum flour

### 3.4 Establishment and evaluation of the design space

Key parameters that had been demonstrated to affect the sorghum flour quality were used to construct the design space. All the parameters for both the ultrasonication processes are listed in Table 4 to illustrate the range of each variable. As long as each variable is maintained within its range, the antioxidant properties (TPC and AA) of the sorghum flour can then be successfully predicted and controlled.

**Table 4:** Range of the variables for guaranteed and successful prediction using the model developed.

	Factors						
	FWR, %w/v		UI, W/cm^2^		UT, s		FR, ml/s
	CU	BU	CU	BU	CU	BU	CU
Lower limit	10	10	20	30	110	195	4
Upper limit	13.5	11.5	25	34	150	212	22

CU: continuous flow ultrasonication; BU: batch ultrasonication

### 3.5 Effect of process parameters on specific energy for batch and continuous flow ultrasonication

**Fig. 4:**
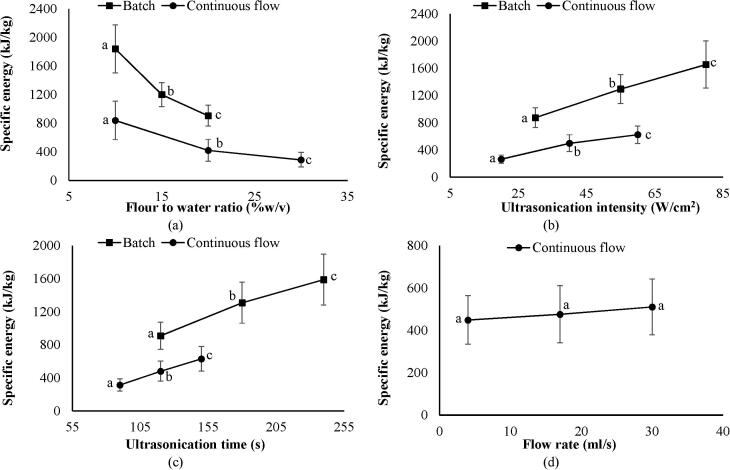
Impact of (a) flour to water ratio, (b) ultrasonication intensity, (c) ultrasonication time and (d) flow rate on specific energy for batch and continuous flow ultrasonication. Values with the different letters at different points in the same line are significantly (p < 0.05) different.

The specific energy was recorded in the range of 624–2520 kJ/kg for the batch ultrasonication, whereas, the continuous flow process exhibited specific energy from 153 to 1158 kJ/kg under their respective process conditions. For both the ultrasonication processes, specific energy significantly (p < 0.05) depleted with an increase in flour to water ratio. However, this diminution was observed higher (65%) in the continuous flow as compared with the batch process (51%) (Fig. 4a). On the other hand, for both the ultrasonication processes, specific energy increased significantly (p < 0.05) with an increase in ultrasonication intensity and time (Fig. 4b, c). As in case of flour to water ratio, similarly, the increment in specific energy was higher (136%, 100%) during the continuous flow when compared with the batch ultrasonication (89%, 75%) with the effect of ultrasonication intensity, and time, respectively. On the contrary, there was no significant (p > 0.05) change was observed in the specific energy when the flow rate increased during the continuous flow ultrasonication (Fig. 4d). All these outcomes immensely support the results obtained in Fig. 1, Fig. 2, Fig. 3. Overall, it could be concluded that the continuous flow ultrasonication provided the sorghum flour with higher TPC and AA despite of consuming lesser specific energy as compared to that of the batch ultrasonication.

### 3.6 Effect of specific energy on total phenolic content and antioxidant activity of sorghum flour during batch and continuous flow ultrasonication

**Fig. 5:**
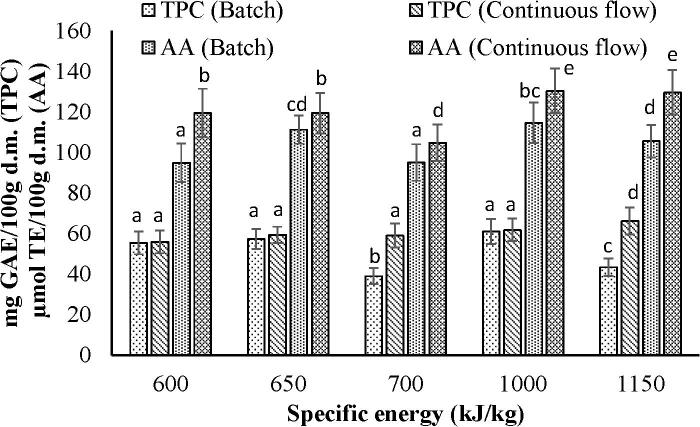
Graph showing the comparative results of total phenolic content and antioxidant activity along with specific energy for batch and continuous flow ultrasonication. Column means within TPC or AA with the different letters are significantly (p < 0.05) different.

Equivalent specific energy was recorded for both batch and continuous flow ultrasonication by varying combinations of experimental parameters within their design space. Flow rate for the continuous flow ultrasonication was used as 17 ml/s. It was observed that for each level of specific energy, the total phenolic content of the sorghum flour treated with the continuous flow ultrasonication was higher than that of treated with the batch process, however, this difference was significant (p < 0.05) beyond 700 kJ/kg (Fig. 5). On the other hand, antioxidant activity of the sorghum flour after continuous flow ultrasonication was observed significantly (p < 0.05) higher than that of obtained from the batch process at each specific energy level (Fig. 5). Again, it could be concluded that specific energy equivalent to the batch process could be obtained during the continuous flow ultrasonication by keeping lower values of ultrasonication intensity, and time and moreover resulting in greater release of phenolics as compared to that of the batch process.

## 4 Conclusions

In the present study, the continuous flow and the batch ultrasonication were used to release the bound phenolics resulted in enhanced TPC and AA in the sorghum flour. Although the maximum TPC and AA was obtained at low ultrasonication intensity with lower concentration of sample for both the processes, the continuous flow ultrasonication released 8% and 7% more TPC and AA, respectively in the sorghum flour at 33% less UI and for 67% higher FWR. Furthermore, the continuous flow ultrasonication released 8% more TPC and almost equivalent AA by consuming 43% and 27% less time, respectively with compared to the batch process. As far as flow rate was concerned for the continuous flow process, moderate flow rate provided the maximum TPC and AA in the sorghum flour. Developed models for both the ultrasonication processes were adequate and precise with the experimental data. At optimum conditions, the continuous flow ultrasonicated sorghum flour had 11% and 7.9% more TPC and AA, respectively than that of the control sorghum flour. Phenolic characterization revealed that salicylic acid followed by ferulic, hydroxybenzoic and caffeic acids mainly contributed to the TPC of the sorghum flour. It was proven that in order to release a higher amount of phenolics, less specific energy was required during the continuous flow as compared to the batch ultrasonication. Moreover, as compared to the batch ultrasonication, lower ultrasonication intensity, and time were required during the continuous flow process to consume similar specific energy. Feasibility of the process at industrial scale can be enhanced by including some pretreatment methods, i.e. fermentation, malting prior to the ultrasonication to improve the extraction of phenolics.

## CRediT authorship contribution statement

**Umesh C. Lohani:** Conceptualization, Methodology, Visualization, Investigation, Writing - review & editing. **K. Muthukumarappan:** Supervision.

## Declaration of Competing Interest

The authors declare that they have no known competing financial interests or personal relationships that could have appeared to influence the work reported in this paper.
